# Cluster-randomised trial to evaluate the ‘Change for Life’ mass media/ social marketing campaign in the UK

**DOI:** 10.1186/1471-2458-12-404

**Published:** 2012-06-06

**Authors:** Helen Croker, Rebecca Lucas, Jane Wardle

**Affiliations:** 1 Health Behaviour Research Centre, Department of Epidemiology and Public Health, University College London, Gower Street, WC1E 6BT, London, UK; 2 Department of Psychology, Royal Holloway University of London, London, UK

## Abstract

**Background:**

Social marketing campaigns offer a promising approach to the prevention of childhood obesity. Change4Life (C4L) is a national obesity prevention campaign in England. It included mass media coverage aiming to reframe obesity into a health issue relevant to all and provided the opportunity for parents to complete a brief questionnaire (‘How are the Kids’) and receive personalised feedback about their children’s eating and activity. Print and online C4L resources were available with guidance about healthy eating and physical activity. The study aims were to examine the impact of personalised feedback and print material from the C4L campaign on parents’ attitudes and behaviours about their children’s eating and activity in a community-based cluster-randomised controlled trial.

**Methods:**

Parents of 5–11 year old children were recruited from 40 primary schools across England. Schools were randomised to intervention or control (‘usual care’). Basic demographic data and brief information about their attitudes to their children’s health were collected. Families in intervention schools were mailed the C4L print materials and the ‘How are the Kids’ questionnaire; those returning the questionnaire were sent personalised feedback and others received generic materials. Outcomes included awareness of C4L, attitudes to the behaviours recommended in C4L, parenting behaviours (monitoring and modelling), and child health behaviours (diet, physical activity and television viewing). Follow-up data were collected from parents by postal questionnaire after six months. Qualitative interviews were carried out with a subset of parents (n = 12).

**Results:**

3,774 families completed baseline questionnaires and follow-up data were obtained from 1,419 families (37.6%). Awareness was high in both groups at baseline (75%), but increased significantly in the intervention group by follow-up (96% vs. 87%). Few parents (5.2% of the intervention group) returned the questionnaire to get personalised feedback. There were few significant group differences in parental attitudes or parenting and child health behaviours at follow-up. Physical activity was rated as less important in the intervention group, but a significant group-by-socioeconomic status (SES) interaction indicated that this effect was confined to higher SES families. Similar interactions were also seen for physical activity monitoring and child television time; with adverse effects in higher SES families and no change in the lower SES families. Effects were little better in families that completed the questionnaire and received personalised feedback. At interview, acceptability of the intervention was modest, although higher in lower SES families.

**Conclusions:**

The C4L campaign materials achieved increases in awareness of the campaign, but in this sample had little impact on attitudes or behaviour. Low engagement with the intervention appeared a key issue.

**Trial registration number:**

Current Controlled Trials ISRCTN00791709.

## Background

Childhood obesity is a major public health issue; data from the Health Survey for England indicated that in 2009, 16.1% of boys and 15.3% of girls aged 2–15 years were obese [1]. In the government publication ‘Healthy Weight, Healthy Lives: a Cross-Government Strategy for England’ [2], reducing childhood obesity was a major focus. A national marketing campaign (Change4Life; C4L) for the primary prevention of childhood obesity was launched in January 2009, initially due to run until 2011. It aimed to encourage target audiences to be aware that overweight has long-term health consequences, recognise that their own family could be at risk, and take responsibility for reducing that risk by adopting healthier family behaviours. C4L used a social marketing approach comprising universal and targeted messages, Initially, the primary target was families with children aged 0–11 years and pregnant women; particularly those from ‘at risk’ groups. The latter were identified during the background consumer insight research as exhibiting behaviours and attitudes towards diet and physical activity that could increase the risk of children becoming obese, and were primarily from lower socioeconomic status (SES) backgrounds [3,4].

Social marketing (SM) interventions use approaches from commercial marketing; typically including advertising, but other marketing strategies as well. They differ from commercial marketing in that they aim to improve the health of individuals and society, rather than bring benefit to those undertaking the marketing [5]. A number of steps for developing interventions have been identified, which include, but are not limited to, basing interventions on theory and carefully considering the needs of the ‘target’ audience [6,7]. SM has been used to promote a variety of health behaviours, including alcohol and drug use, seat belt use, oral health, smoking, safe sex, and drink-driving [6,8], and a review of the field found evidence of beneficial effects [5].

Given the scale of obesity, SM interventions have obvious appeal based on their wide reach. A review of childhood obesity prevention programmes found that SM techniques were being used more frequently [9], but there was no evidence that they improved programme success; although the authors speculated that this was because they were used inconsistently and may have been under-reported. The VERB campaign is probably the most widely reported; set in the US, it targeted children aged 9–13 years and used SM to increase physical activity [10]. Children’s awareness of the campaign was associated with increased physical activity [10] and effects persisted in the long-term [11]. Another campaign targeting parents of inactive children in Canada resulted in higher campaign awareness; this was associated with knowledge and saliency around physical activity, but actual behaviour was not measured [12]. However, if positive effects are restricted to children who are aware, it is impossible to rule out reverse causation; i.e. that children who are interested in healthy changes are more likely to notice relevant campaigns. Analyses comparing matched exposed and non-exposed populations are needed to establish causation, but these are difficult to achieve.

In the UK, there have been many local SM campaigns to tackle obesity, nutrition or exercise (http://thensmc.com/resources/showcase/by-subject.html); but few evaluations have been published. ‘Snack Right’ was a local campaign targeted at parents to change the snacking habits of pre-school children [13]. Results indicated that although parents reported spending more on fruit (not vegetables), children’s intake did not increase, nor did children’s overall consumption of snacks change, although parents reported that their children had eaten fewer sugary foods and drinks. An earlier study evaluated an educational campaign, ‘Fighting Fat, Fighting Fit’ (FFFF), run by the BBC and directed at adults. Evaluation of FFFF showed high awareness and recall of messages, particularly among those from higher SES groups [14], and in a sub-sample participating in a pre-post evaluation study, significant changes were reported in fat and fruit and vegetable intake, physical activity and weight. However, as there was no control condition, the changes could not be attributed to the campaign [15].

Although challenging, it is crucial to evaluate social marketing initiatives, both to inform future programme development and to establish value for money with use of public funds [16]. With this in mind, the Department of Health commissioned the current study to independently evaluate C4L. The specific aim was to evaluate the impact of the ‘family information pack’ element of C4L, using a randomised, controlled study design, on (i) parents’ attitudes to their children’s eating, activity and weight, (ii) their intentions to change eating and activity behaviours and (iii) the reported diet and activity behaviours of parents and children.

## Methods

### Description of the social marketing intervention

The campaign has been outlined in detail [3], but is described briefly here. The specific aims were to encourage the target groups to: i) be aware of the health risk of excess body fat, ii) reduce calorie intake and develop healthier eating habits (reductions in foods high in added sugar and fat, a more regular meal pattern, less snacking, and increased fruit and vegetable intake), and iii) participate in regular physical activity (especially family activities) and reduce sedentary time. The campaign was launched in England in January 2009 with television, print and poster advertising, a helpline, a website (http://www.nhs.uk/change4life/Pages/change-for-life.aspx), and accompanying material resources. The term ‘obesity’ was specifically not mentioned in any materials.

There were four phases to the campaign. Phase 1 aimed to ‘reframe’ obesity as a health rather than an appearance issue; and one that was relevant to everyone. Phase 2 encouraged families to engage with the campaign by completing the ‘*How are the Kids*’ (HTK) questionnaire. From this they would receive feedback in the form of a personalised ‘family information pack’ (Phase 3). The HTK questionnaire and personalised family information pack were based on the campaign’s eight targets for child behaviour change (reducing intake of fat (especially saturated fat), reducing sugar, controlling portion size, consuming at least five portions of fruit and vegetables a day, having a regular pattern of three meals per day, reducing snacking, doing at least an hour of moderate-intensity activity per day, and reducing sedentary time). Phase 4 supported ‘at risk’ families (primarily lower SES) with regular booster materials by mail or on-line. A parallel broader aim was to create ‘societal movement’ which would lead to a more supportive environment within which families could make changes, by involving partner organisations including schools, health professionals, councils, charities, workplaces, and supermarkets. The specific role of these stakeholders was not defined, but involved encouragement for families to participate in the campaign. The current research project focused on Phase 2 (access to the print resources and completion of the questionnaire) and Phase 3 (receiving the family information pack).

### Design of evaluation study and participants

The study ran from summer 2009 to summer 2010, as such the study was carried out during implementation of the national campaign. Parents^a^ were recruited from 40 state-funded primary schools across England selected to represent a mix of school types (faith vs. non-faith), demographic characteristics (family SES and ethnicity), urban and rural areas, and a wide geographical spread. Recruitment was carried out in two waves; with parents of all children in school years one to six (ages 5–11 years) receiving an invitation to participate in the study by returning a brief baseline questionnaire and providing contact details for the research team. The study information indicated that it was about children’s health and development, but C4L was not specifically mentioned.

Randomisation was carried out by a statistician and researchers enrolled participants. Schools were randomised to intervention or ‘usual care’ (control) using constrained randomisation to take account of variation in school size. They were ranked by school size and this was used as a blocking factor; each block comprised two schools and within blocks, schools were randomly allocated to intervention or control conditions. The control group had standard exposure to healthy lifestyle messages (for example, national healthy eating guidelines and ‘5-a-day’ messages). Parents in the intervention group were sent the C4L materials available as part of the national campaign and the HTK questionnaire. Those returning the HTK questionnaire were sent the ‘family information pack’ which included personalised feedback relating to their family’s eating and activity behaviours. Due to the nature of the intervention, it was not possible to blind participants to group allocation. To ensure that all parents in the intervention group were exposed to a higher ‘dose’ of C4L materials, those in the intervention group who did not complete the HTK questionnaire were sent a generic version of the ‘family information pack’ without the personalised element. It was considered appropriate to amend the study protocol in response to non-participation with the HTK questionnaires since this reflected typical population-based behaviour, hence was a realistic evaluation of how the campaign was received by families in the UK. This deviation from the study protocol was implemented during the study; it was not planned a priori, but was agreed by the study team and Department of Health. All parents were sent a follow-up questionnaire after six months, with two reminders if it was not returned.

Qualitative home-based interviews were carried out with a subset of parents (n = 12) in the intervention group, selected to ensure representation from lower and higher SES families. The interviews aimed to explore families’ reactions to C4L, their lifestyle changes and the reasons for change or not. Respondents were also asked about the key messages they had taken from the campaign, how the materials were used, and what a ‘healthy lifestyle’ meant to them.

Ethical approval was granted by the University College London Research Ethics Committee.

### Measures included in evaluation study

#### Demographic, anthropometric and attitudinal characteristics at baseline

Parents reported their ethnicity, age, highest level of education, child’s date of birth and their relationship to the child. Parental education used as the measure of SES [17]. Categorical variables were dichotomised for analysis: white vs. non-white for ethnicity, and university vs. non-university for education. Parents reported their weight and height, from which BMI was calculated and weight status was determined using World Health Organisation cut-offs [18]. Perception of the child’s weight status was assessed using the question ‘How would you describe your child’s weight at the moment’, with five response options (very underweight, slightly underweight, average weight, slightly overweight or very overweight).

Attitudes towards the child’s diet and physical activity were assessed with four questions. First parents were asked to rate the importance of a healthy diet and adequate physical activity for their child’s health, with responses on five-point Likert scales from ‘not at all’ to ‘extremely’. They also evaluated their child’s current diet and level of physical activity, with three response options for each; ‘yes my child eats healthily/ does enough physical activity at the moment’, ‘no I would like him/her to eat a little more healthily/ do a little more activity’, or ‘no I would like him/her to eat a lot more healthily/ do a lot more activity’

#### Awareness of the campaign

Awareness of C4L at baseline and follow-up was assessed by asking parents whether they had heard of the campaign, with yes/no response options. Awareness of a new initiative (Start4Life), designed for a younger age group and launched after C4L, was additionally included at follow-up. Questions on a number of other health promotion initiatives were asked as distracters.

#### Outcome measures at follow-up

The study included a range of attitudinal and behavioural outcomes relating to children’s eating and activity behaviours, as outlined below. Validated measures were used where possible but the majority were created for the study. Although included in the initial protocol as a study outcome, parents’ knowledge about food and activity recommendations was not included in order to minimise participant burden and maximise recruitment. A summary of the outcomes included and how these relate to the C4L targets is provided in Table 1 and more detailed information regarding questionnaire scoring is available in the Additional file 1: Table S1.

**Table 1:** Summary of outcome measures used in the study

**Key targets in C4L**^**1**^	**Outcome measures used in evaluation study**^**2**^		
	**Attitudinal and intention to change**^**3**^	**Child behaviour**^**4**^	**Parent behaviour**^5^
Reduce fat intake (especially saturated)	Snack mainly on healthy foods	Snacks	Keeping track of snack foods (e.g. crisps, cheesy crackers)/ high-fat foods
	Limit high-fat foods eaten at mealtimes		
Reduce added sugar intake	Snack mainly on healthy foods	Sugary drinks	Keeping track of sweet things (e.g. sweets, ice-cream, cake, biscuits, chocolate)
	Limit number of sugary drinks	Snacks	
	Avoid sugary breakfast cereals		
Control portion size	Not to eat too much food		
Achieve 5 portions of fruit and vegetables per day	Eat 5 or more portions of fruit and vegetables	Fruits	Modelling of eating healthy foods
		Vegetables	
3 regular mealtimes per day	Eat 3 meals at regular times		
Reduce snacking	Eat no more than 2 snacks	Snacks	
At least 1 hour of moderate intensity physical activity per day	Spend at least 60 minutes being active	Number of days child is physically active for at least 60 minutes	Keep track of child physical activity Modelling of being active
Reduce sedentary time	Limit time spent watching TV/ on computer	Hours of TV/ video/ computer on a typical weekday/ weekend day	

^1^As described in ref 3; ^2^These are the outcome measures included in the questionnaire at follow-up, details of questionnaire scoring are provided in the Additional file 1: Table S1; ^3^Parents’ attitudes to their child’s behaviour each day (comprised ratings of importance and ease of carrying out each behaviour) and intention to change; ^4^Frequency of consumption of each food or of child doing 60 minutes of physical activity in a day; ^5^Parent rating of these behaviours.

#### 1. Attitudinal outcomes

To facilitate parents’ answering questions, ten specific behaviours relating to children’s eating and activity were selected to map onto the key C4L messages. These comprised eight dietary behaviours and two physical activity behaviours (outlined in Table 1). Parents were asked to rate how important and how easy it was to achieve each behaviour. Higher scores indicated greater importance and ease.

#### 2. Intention to change

Parents were asked whether they intended to encourage their child to do these ten behaviours over the following three months. Higher scores indicated greater intention.

#### 3. Behavioural outcomes: child dietary and activity behaviours

To minimise questionnaire burden it was not possible to include a full food frequency questionnaire or to use diet diaries, instead four foods were selected as indicators of children’s dietary intake (outlined in Table 1). An overall healthy eating score was calculated using the mean of the scores with a higher score indicating healthier eating. Again to minimise participant burden, child’s physical activity and sedentary behaviour were measured using single items. For analysis, an average of the daily hours of activity and of the daily hours of media viewing was calculated.

#### 4. Behavioural outcomes: parenting behaviours

Monitoring of the child’s eating was assessed with the monitoring subscale of the ‘Child Feeding Questionnaire’ (CFQ); a validated measure of child feeding [19]. Monitoring of physical activity was assessed with a question adapted from the PACE physical activity measure for adolescents (PACE, 2001: San Diego State University, http://www.paceproject.org/Measures.html). Modelling of healthy dietary behaviours was assessed with the modelling subscale of the ‘Comprehensive Feeding Practices Questionnaire’ (CFPQ) [20]. These questions were adapted for physical activity, using the same wording and response options but asking about ‘being active’ rather than ‘eating healthy foods’. Higher scores indicated more monitoring and modelling.

Test-retest validation for the questions about child behaviour and physical activity modelling (since these were not from validated questionnaires) was conducted in a separate convenience sample of parents (n = 54). Correlations between responses for the dietary behaviours were all >0.75, apart from the question relating to sugary drink intake (r = 0.69). The correlation for the questions about TV watching was 0.80 and for hours of physical activity was 0.71. The correlation for the questions relating to modelling of physical activity was 0.86. This indicated that the questions performed adequately.

### Sample size calculation

We based this on the monitoring scale of the CFQ [19] since it has been validated for the age group in the study and reference data were available in similar populations. The sample size calculation assumed a difference between the means in the intervention and control groups of 0.33 (effect on mean of an upward shift on one item), a s.d. of 0.84 obtained from a previous study in a similar sample [21], and 35 children being recruited and fully participating in the study per school. Based on a relatively conservative estimated intra-class correlation (ICC) of 0.15, 20 schools per group were required, using a power of 80% for a two-tailed test with a critical value of p = 0.05. The actual ICC in the study was 0.07 so the study was well powered.

### Statistical analysis

Data were analysed using the Statistical Package for the Social Sciences (SPSS) version 18 (SPSS Inc, Chicago, IL). Several families had more than one child in the same school, and to avoid duplication of responses, parents were asked to complete the questionnaire for the older child. Baseline characteristics were compared in those returning (responders) and not returning (non-responders) the follow-up questionnaire. Independent samples t-tests were used for continuous outcomes and chi-squared tests for categorical outcomes.

The main analyses were carried out on the sample of families for whom follow-up data were available (responders). Differences between randomisation groups in awareness of C4L were tested at baseline and follow-up and of Start4Life at follow-up, using chi-squared tests. For attitudinal and behavioural outcomes, complex samples general linear model (GLM) analyses were used to test for between group differences and interactions with SES with adjustment for clustering by school. There were six attitudinal outcomes (importance, ease and intention for healthy child diet and activity behaviours), four parenting outcomes (monitoring and modelling of healthy eating and physical activity), and three child behaviour outcomes: overall healthiness of diet (composite score of snack, fruit, vegetable, and sugary drink intake), physical activity (days per week active), and television viewing (hours per day). Randomisation group and parent education (university vs. non-university) were included as fixed factors. Covariates included in all models were parent age, BMI, and ethnicity, and child age and gender. Baseline levels were included as covariates where there were similar items, but these were not available for monitoring or modelling. Outcomes and covariates were assumed to be linear, apart from covariates which were categorical (child gender) or which had been dichotomised for analysis (ethnicity and education).

Further analyses were conducted to examine the impact of C4L materials on families who actively participated in the campaign by returning the HTK questionnaire. Complex samples GLM analyses were repeated as described above, but with three groups: ‘engaged intervention’, ‘non-engaged intervention’ and control.

## Results

### Participants

**Figure 1:**
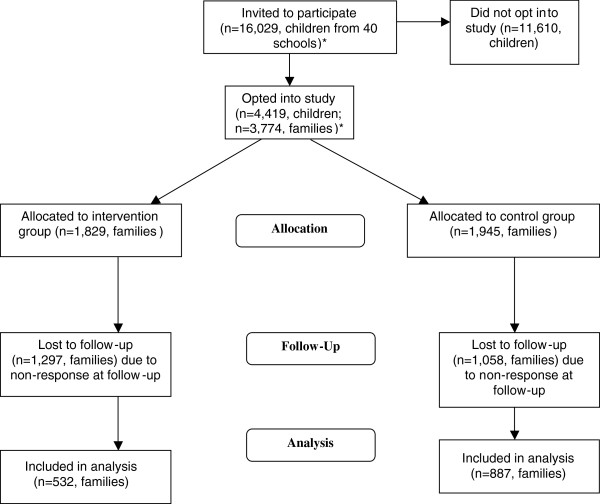
Participant flow through trial.*Several families had more than one child in the same school, and to avoid duplication of responses, parents were asked to complete the questionnaire for the older child. This was not known at baseline; subsequent analyses included only one child per family hence numbers are presented for families rather than individual children.

Baseline characteristics of the sample are outlined in Table 2 and a flowchart of participants through the study is shown in Figure 1. A total of 16,029 children were given invitation letters at their schools; 3,774 families with 4,419 children returned the baseline questionnaire (28% response rate). Most questionnaires (88%) were completed by mothers. Around three quarters of the families were white (76%, n = 2831) and there were similar numbers of girls (49%) and boys (51%). Approximately a third of parents were educated to university level (35%, n = 1300). Children were on average 8.3 (s.d. = 1.8) years old, and parents were 38.3 (6.2) years. Parents’ mean BMI was 24.9 (4.6), with 40% (n = 1392) overweight or obese. The majority of parents described their child’s weight as ‘average’ (74%, n = 2788) and only 11% (n = 414) as ‘slightly’ or ‘very overweight’.

**Table 2:** Baseline characteristics of the sample

**Mean (s.d.) unless stated**		**Whole sample**		**Responders only**^**§**^	
	**Non-Responders (n=2355)**	**Responders (n=1419)**	**Group difference**	**Intervention group (n=532)**	**Control group (n=887)**
Child age (years)	8.25 (1.85)	8.34 (1.83)	t(3707)=-1.5, p=0.01	8.23 (1.86)	8.42 (1.80)
Parent age (years)	37.44 (6.23)	39.72 (6.01)	t(2979)=-10.9, p<0.001**	38.99 (6.18)	40.16 (5.87)
Parent BMI	25.14 (4.71)	24.45 (4.41)	t(3035)=4.3, p<0.001**	25.12 (4.98)	24.05 (3.98)
Child gender, n (%)					
Male	1189 (50.6)	717 (50.6)	X^2^(1)=0.0, p=1.0	268 (50.4)	449 (50.7)
Female	1160 (49.4)	700 (49.4)		264 (49.6)	436 (49.3)
Ethnicity, n (%)					
White	1667 (75.7)	1164 (82.7)	X^2^(1)=59.1,	422 (79.8)	742 (84.4)
Non-white	663 (24.3)	244 (17.3)	p<0.001**	107 (20.2)	137 (15.6)
Parent education, n (%)					
University	695 (35.2)	605 (43.2)	X^2^(1)=62.0,	177 (33.8)	428 (48.7)
Non-university	1592 (64.8)	797 (56.8)	p<0.001**	347 (66.2)	450 (51.3)
Importance diet (1-5)^+^	4.50 (0.75)	4.61 (0.63)	t(3389)=-4.8, p<0.001**	4.58 (0.68)	4.63 (0.59)
Importance activity (1-5)^+^	4.47 (0.71)	4.56 (0.60)	t(3376)=-3.9, p<0.001**	4.52 (0.67)	4.58 (0.55)
Ease diet (1-5)^+^	3.67 (0.96)	3.62 (0.97)	t(3709)=1.5, p=0.1	3.66 (1.01)	3.60 (0.95)
Ease physical (1-5)^+^	3.90 (0.89)	3.81 (0.92)	t(2856)=3.0, p=0.003**	3.84 (0.90)	3.79 (0.93)
Rating of diet adequacy, n (%)					
Yes	1426 (63.2)	956 (67.5)	X^2^(1)=17.9, p<0.001**	363 (68.4)	593 (67.0)
No	925 (36.8)	460 (32.5)		168 (31.6)	292 (33.0)
Rating of activity adequacy, n (%)					
Yes	1605 (70.2)	1040 (73.4)	X^2^(1)=11.3,	388 (73.2)	652 (73.6)
No	746 (29.8)	376 (26.6)	p=0.001**	142 (26.8)	234 (26.4)

**p < 0.01; ^**§**^Sample used for main analyses; ^+^Higher score indicates a higher rating of importance or ease.

A total of 1,419 families returned follow-up questionnaires; 38% of the recruited sample and 12% of those initially invited to participate. Compared with parents who did not respond at follow-up, those who returned this questionnaire were older, had a lower BMI, and were proportionally more white and better educated. They also rated diet and activity to be marginally more important at baseline and were more likely to regard their child’s diet and activity to be adequate, but found it less easy to help their child to be active. There were substantial differences between randomisation groups for ethnicity and education (see Table 2) with parents in the intervention group being less well-educated and proportionally less white.

### Awareness of campaign

As shown in Table 3, the majority (75%) of families in both groups were aware of C4L at baseline. At follow-up, awareness had increased in both groups, but more so in the intervention group (to 96% vs. 87%). More families in the intervention group had also heard of Start4Life at follow-up (54% vs. 43%) suggesting that attention to the campaign had generalised.

**Table 3:** Awareness of the campaign

	**Intervention group n (%) aware**	**Control group n (%) aware**	**Group difference**
**Change4Life:**			
Baseline	391 (75.3)	661 (75.5)	X^2^(1) = 0.003, p = 1.0
Follow-up	508 (96.4)	757 (86.8)	X^2^(1) = 34.8, p < 0.001**
**Start4Life:**			
Follow-up	259 (50.6)	370 (43.0)	X^2^(1) = 7.5, p = 0.006**

**p < 0.01.

### Post-intervention comparison: overall effects

**Figure 2:**
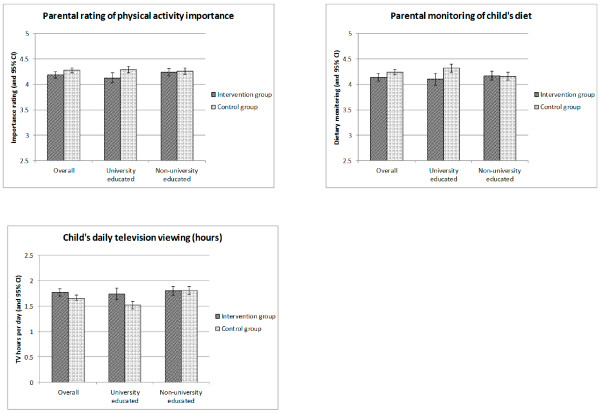
PA importance/ dietary monitoring/ TV hours in the intervention vs. control group.

These results are outlined in Table 4 and Figure 2. There was a significant overall effect of the intervention for physical activity importance only, with the intervention group giving significantly *lower* ratings at follow-up than the control group (mean and 95% CI of 4.19 (4.12, 4.25) and 4.28 (4.23, 4.32) for the intervention and control groups respectively).

### Post-intervention comparison: SES interactions

These results are outlined in Table 4 and Figure 2. There was a near-significant interaction between SES and group for rating of physical activity importance, with adverse effects of the intervention only being seen for the higher SES families, and virtually no group differences in the lower SES families. There were also significant interactions with SES for dietary monitoring and TV hours showing the same pattern, with the intervention group doing worse than the control group in the higher SES group and no differences in the lower SES group.

**Table 4:** Post-treatment attitudes and behaviour in the intervention and control groups (by social class)

	**Mean (95% CI)**				**R**^**2**^	**Overall treatment effect**	**Interaction effect**
	**Intervention group**		**Control group**			**Wald F (df1,df 2), p**	**Wald F (df1,df 2), p**
	**University education (n=177)**	**Non university education (n=347)**	**University education (n=428)**	**Non university education (n=450)**			
**Importance (1-5)**							
Diet	4.15 (4.07, 4.24)	4.28 (4.21, 4.34)	4.27 (4.22, 4.33)	4.26 (4.21, 4.32)	0.07	F(1,39)=2.3, p=0.14	F(1,39)=2.7, p=0.11
PA	4.13 (4.03, 4.23)	4.24 (4.17, 4.32)	4.29 (4.23, 4.36)	4.26 (4.20, 4.33)	0.07	F(1, 39)=5.1, p=0.03*	F(1, 39)=3.5, p=0.07
**Ease (1-5)**							
Diet	3.91 (3.80, 4.01)	3.82 (3.74, 3.89)	3.94 (3.87, 4.01)	3.74 (3.67, 3.81)	0.15	F(1, 39)=0.2, p=0.6	F(1, 39)=2.0, p=0.17
PA	3.50 (3.36, 3.64)	3.65 (3.54, 3.75)	3.54 (3.44, 3.63)	3.57 (3.48, 3.66)	0.10	F(1, 39)=0.08, p=0.78	F(1, 39)=0.8, p=0.40
**Intention (1-5)**							
Diet	4.28 (4.19, 4.38)	4.27 (4.20, 4.34)	4.37 (4.30, 4.43)	4.27 (4.21, 4.34)	0.03	F(1, 39)=1.6, p=0.22	F(1, 39)=0.8, p=0.37
PA	4.12 (4.00, 4.24)	4.06 (3.97, 4.15)	4.18 (4.10, 4.26)	4.01 (3.93, 4.09)	0.03	F(1, 39)=0.02, p=0.9	F(1, 39)=1.2, p=0.28
**Monitoring (1-5)**							
Diet	4.10 (3.98, 4.22)	4.17 (4.09, 4.26)	4.32 (4.24, 4.39)	4.16 (4.08, 4.23)	0.06	F(1, 39)=2.4, p=0.13	F(1, 39)=6.7, p=0.01*
PA	3.43 (3.26, 3.60)	3.48 (3.35, 3.61)	3.56 (3.45, 3.67)	3.34 (3.23, 3.46)	0.03	F(1, 39)=0.0, p=0.98	F(1, 39)=3.5, p=0.07
**Modelling (1-4)**							
Diet	3.60 (3.52, 3.68)	3.51 (3.45, 3.57)	3.59 (3.54, 3.64)	3.54 (3.49, 3.59)	0.03	F(1, 39)=0.1, p=0.70	F(1, 39)=0.5, p=0.48
PA	3.29 (3.19, 3.40)	3.25 (3.17, 3.33)	3.29 (3.23, 3.36)	3.30 (3.23, 3.37)	0.05	F(1, 39)=0.3, p=0.57	F(1, 39)=0.6, p=0.46
**Behaviour**							
Diet score (1-7)	4.89 (4.77, 5.01)	4.70 (4.61, 4.79)	4.98 (4.90, 5.06)	4.63 (4.55, 4.71)	0.18	F(1, 39)=0.04, p=0.84	F(1, 39)=3.2, p=0.08
PA (d/wk active)	5.60 (5.40, 5.80)	5.48 (5.33, 5.63)	5.48 (5.34, 5.61)	5.37 (5.24, 5.50)	0.12	F(1, 39)=1.8, p=0.19	F(1, 39)=0.008, p=0.93
TV (hrs/day)	1.73 (1.61, 1.85)	1.81 (1.72, 1.90)	1.52 (1.44, 1.60)	1.80 (1.73, 1.88)	0.10	F(1, 39)=3.2, p=0.08	F(1, 39)=4.2, p=0.046*

*p < 0.05; analyses are complex samples GLM, with adjustments made for school clustering, and interactions with SES; post-intervention values are adjusted for baseline equivalents where available, parent age, BMI and ethnicity, and child age and gender; a higher score indicates a higher level of attitude or behaviour for all outcomes.

### Qualitative feedback

A summary of the feedback from the qualitative interviews is presented in Table 5; quotes are grouped to reflect the main outcomes of the study. Regardless of SES, parents tended to be satisfied with their families’ eating and activity behaviours and to consider change unnecessary. There was some acknowledgement among lower SES parents that their own eating and activity habits were ‘not perfect’ although they considered their children’s behaviour to be healthy. Few parents said that they monitored their children’s eating or activity and some lower SES parents thought this was unrealistic, while some higher SES parents felt they already did it. There were also few comments specifically relating to diet or activity, but these suggested that the cost of healthier eating was seen as a barrier by lower SES families, although one parent had found that ‘scratch cooking’ was more economical. There were varying views about the campaign itself with several families, lower and higher SES, considering that they already did the recommended behaviours, and one parent feeling that the recommendations were unrealistic. Most parents were positive about the materials themselves, especially those aimed at children, although some higher SES parents considered them patronising.

**Table 5:** Qualitative feedback regarding the campaign

**Topic**	**Quotes**
**Family lifestyle**	“I think we’re not too bad really… I can’t think of anything [that I would like to change]” (lower SES, ID 3067)
	“It’s been fairly easy for us to lead a lifestyle that’s relatively healthy [according] to what they [C4L materials] say and what I see other people do” (lower SES father, ID 3047)
	“We eat quite healthily.. quite happy with the amount of activity we do” (lower SES, ID 4012)
	“I’ve always tried [to help] them to get their exercise and their five a day” (lower SES, ID 1287)
	“I’m quite happy with what they [children] are doing, I don’t think we could fit much more in” (higher SES, ID 3054)
**Modelling**	“I don’t do enough exercise but the children do… I’m a bit lazy” (lower SES, ID 1287)
	“I’m probably the worst… my bad [snacking] habits bring the others down” (lower SES father, ID 3047)
	“I could do with eating some more fruit… I should really eat a couple of pieces a day… what I should do is instead of having a biscuit when she’s having some fruit I should have some fruit as well. I know I should really change it [but] you get into a habit and it’s hard to break out of that habit” (lower SES, ID 1199)
**Monitoring**	“I looked at [the C4L materials] and I thought ‘yeah that’s really good’ but then I just don’t have the time or the inclination to start [filling in the food charts]… part of me [didn’t] want to see what the results would be… I know what we are eating and don’t need to log it ” (lower SES, 3113)
	“We do make sure that at the weekends we’re out and about and we’re always thinking about how much exercise he’s had” (higher SES, ID 1054)
**Dietary intake**	“I use the ‘traffic light system’ [on food labels] to pick out healthy cereals … I just did it [made the changes] and no-one noticed … it wasn’t very difficult” (lower SES, ID 190)
	“The cost comes into it … if you make a batch of soup it’s a lot cheaper than buying tinned stuff and tastes better … and the salt content in a lot of soups is high” (lower SES, ID 190)
	“I should try a bit harder to do some healthy snacks … but it’s quite often more expensive having the healthier snacks” (lower SES, ID 140)
	“Coco Pops and Frosties do have a lot of sugar in them” (lower SES, ID 4012)
	“I did read somewhere that sugary cereals aren’t good … but they [children] tend to eat Coco Pops, Cheerios … there must be something good in them … and they’re drinking milk as well” (lower SES father, ID 3047)
**Change for Life**	“Positive reinforcement and reminds you that you are doing the right thing” (lower SES, ID 140)
	“Some of it I did look at and some of it I didn’t … I didn’t pay too much attention to it because hopefully I’m doing the right thing anyway ” (lower SES, ID 1287)
	"These things [8 C4L behaviours] are unrealistic for mums that work full time!" (lower SES, ID 3138)
	“This is a little patronising. It is easy to be in control of what my child eats as I buy and cook the food. I don't need to actively encourage him to be active - he just is! Plus I am the adult and can limit how much computer/TV he watches. All things in moderation - I don't want him obsessed with weight or the way he looks. You live what you learn." (higher SES, ID 1596)
	"It [C4L materials] was stuff I already know, so I must admit I binned it, I felt a little bit patronised and thought 'I know this, I don't need to be told' but I realise that lots of other people do" (higher SES, ID 1054)
	“We had a big pack with stickers in which xxx found absolutely fascinating… [I] had a quick read when they [materials] arrived but they got put in the bin after a while” (higher SES, ID 3054)
	“Stickers, the calendar, there was snap cards… we loved those” (higher SES, ID 2421)
	“When I first looked at it I thought ‘oh God, how am I supposed to read all this’ but it was all sort of ‘bite size’” (higher SES, ID 2421)
	“I got it and quickly flicked through it and then I put it aside and didn’t go back to it. Obviously it is there in case I need to look back” (lower SES, ID 190)
	“Thought it was very good and I think it’s very colourful and bright as well and I think that engages them” (lower SES, ID 3067)

### Sub-analyses on families who actively participated in C4L

98 families filled in and returned the ‘How are the Kids’ questionnaire and were deemed to have actively participated in C4L (2.6%); 95 were in the intervention group and only three in the control group. Overall 5.2% of the intervention group actively enrolled, of whom 52 (9.8% of the total) also completed their follow-up questionnaires and were included in this analysis. Baseline characteristics of the engaged and non-engaged sub-groups of the intervention group are shown in Table 6. In the intervention group, parents who returned the HTK questionnaire (the ‘engaged intervention’ group) were younger, rated it easier to help their child to eat healthily, and were less likely to have had a university education compared to those who did not return the HTK questionnaire.

**Table 6:** Baseline characteristics of participants in the intervention group (engaged vs. non-engaged)

**Mean (s.d.) unless stated**	**‘Engaged’ (n=52)**	**‘Non-engaged’ (n=480)**	**Group difference**
Child age (years)	7.96 (2.04)	8.26 (1.84)	t(525)=1.09, p=0.28
Parent age (years)	37.06 (5.61)	39.20 (6.21)	t(512)=2.33, p=0.02*
Parent BMI	25.52 (4.33)	25.07 (5.04)	t(510)=-0.61, p=0.55
Child gender, n (%)			
Male	25, 48.1%	243, 50.6%	X^2^(1)=1.22, p=0.73
Female	27, 51.9%	237, 49.4%	
Ethnicity, n (%)			
White	46, 88.5%	376, 78.8%	X^2^(1)=2.70, p=1.00
Non-white	6, 11.5%	101, 21.2%	
Parent education, n (%)			
University	11, 21.2%	166, 35.2%	X^2^(1)=4.11, p=0.04*
Non-university	41, 78.8%	306, 64.8%	
Importance diet (1-5)+	4.58 (0.64)	4.58 (0.68)	t(528)=0.07, p=0.95
Importance activity (1-5)+	4.48 (0.67)	4.53 (0.67)	t(527)=0.48, p=0.63
Ease diet (1-5)+	4.02 (0.83)	3.62 (1.02)	t(69.22)=-3.23, p=0.006**
Ease physical (1-5)+	3.96 (0.79)	3.83 (0.91)	t(519)=-1.02, p=0.31
Rating of diet adequacy, n (%)			
Yes	40, 76.9%	323, 67.4%	X^2^(1)=1.95, p=0.16
No	12, 23.1%	156, 32.6%	
Rating of activity adequacy, n (%)			
Yes	34, 65.4%	354, 74.1%	X^2^(1)=1.80, p=0.18
No	18, 34.6%	124, 25.9%	

*p < 0.05, **p < 0.01; +Higher score indicates a higher rating of importance or ease.

**Figure 3:**
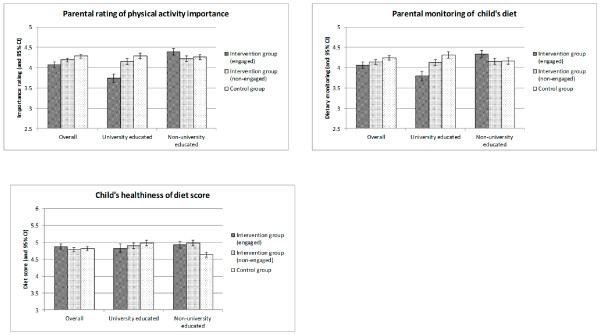
Importance of physical activity, dietary monitoring and healthiness of diet score in the ‘engaged’ intervention vs. ‘non-engaged intervention vs. control group.

Comparing outcomes across the three groups (‘engaged intervention’, ‘non-engaged intervention’ and control), there were no significant intervention effects for any outcomes, but there were significant interactions with SES for parents’ rating of importance of physical activity (Wald F(2,38) = 4.3, p = 0.02), dietary monitoring (Wald F(2,38) = 6.2, p = 0.005), and child diet score (Wald F(2,38) = 5.6, p = 0.007). These are shown in Figure 3. In the higher SES group, scores were lower in the intervention condition, and for the lower SES group there was a tendency for the engaged intervention group to give higher scores. There were no significant interactions with SES for any of the other attitudinal or behavioural outcomes.

## Discussion

This study found that provision of the ‘family information pack’ from C4L resulted in significantly higher awareness of the C4L campaign, as well as for the Start4Life campaign that started at a later date, but disappointingly, there were few positive effects on attitudes or behaviour.

### Summary of results and comparison with other studies

Awareness of C4L, despite being high at baseline, increased more in the intervention group. However active engagement by completing the ‘How are the Kids’ questionnaire was very low. The materials were therefore not prompting active involvement with the campaign. 390,197 families completed HTK questionnaires as part of the national C4L campaign between February 2009 and August 2011 (Data Lateral, London; Personal communication), this equated to 6.5% of families, based on population estimates of families in England with dependent children in 2001 [22]. High campaign awareness is in line with other reports of social marketing interventions targeted at adults [14,23-25] and children [10,12]. The low level of ‘sign-up’ is also in line with other studies. In ‘Fighting Fat, Fighting Fit’ only an estimated 0.2% of the exposed population enrolled in the more intensive aspects [14].

There was only one significant difference between the intervention and control groups at follow-up; the intervention group reported lower levels of the importance of child’s physical activity than the control group. For the attitudinal and behavioural outcomes, higher and lower SES parents appeared to react differently to the intervention. Higher SES parents in the intervention group rated the importance of physical activity lower, and reported less dietary monitoring and TV watching than in the control group, while there were no significant group differences for lower SES parents. Among parents who actively engaged with C4L, the outcomes were similar to the main analyses; the higher SES ‘engaged intervention group’ had more negative scores than the ‘non-engaged intervention’ or the control groups, while for lower SES families there was a trend for more positive scores in the ‘engaged intervention’ than the other two groups.

In contrast to these results, other studies of social marketing campaigns have found positive effects on attitudinal outcomes with increased self-efficacy in adults [24,26] and children [11], and more positive attitudes towards target behaviours in adults [23,26,27] and children [11]. Studies using intention to change as a proxy measure of behaviour change in target behaviours found increased intentions in adults [25,28]. Fewer studies have measured behavioural outcomes and results have been inconsistent; some have found no changes in behaviour in adults [24,27] or children [10], while others have reported positive changes in adults [23], children [12,13][12,13] and parents [29]. Although one campaign (VERB) found no overall increase in children’s physical activity [10], level of activity correlated with campaign exposure [11] and was increased in certain subgroups, including children with low levels of activity at baseline and whose parents had low educational levels and lived in densely populated urban areas [10].

It is unclear why the results were so disappointing for C4L. Only a small proportion of participants actively engaged with C4L, therefore most did not receive personalised feedback, which is likely to have diluted any impact on the intervention group. It is unclear why engagement was so low as feedback could not be obtained from these parents. Another factor could be that C4L targeted multiple complex diet and activity behaviours, whereas many other campaigns have had a narrower focus. There is evidence (in adults) that single component physical activity and dietary interventions are more effective than those targeting multiple behaviours [30]. There is also evidence of this from SM initiatives; VERB and ParticipACTION both focused on physical activity [10,12], and ‘The Food Friends: Making New Foods Fun for Kids’ specifically targeted acceptance of new foods in pre-schoolers [31]. Inclusion of multiple target behaviours may reduce the perception of importance of any single behaviour and could lead people to focus on the ones they feel they are already achieving, and therefore focus less on those which could be improved.

The lack of effect could also be due to lack of clarity over whether the child or the parents were the targets. In another childhood obesity campaign (‘5-4-3-2-1-Go!’) in low income communities, some effects were seen on parents’ behaviour but none on children’s [29]. Given that ultimately parents are being asked to implement change, being more explicit about this in the campaign messages, and measuring proximal targets of parental behaviour change, may increase the likelihood of success. Several parents commented on how engaging the materials were for their children as if they were the intended target. C4L stated that its focus was on families “… the focus will usually be the mother, who is more often the gatekeeper of diet and activity .” [3]. However, C4L also produced materials for children, for example, snap cards and other child-friendly activities which may have distracted parents from the key messages of change in family behaviours.

There could have been other problems with the campaign design. The campaign was based on a hypothetical model of behaviour change, but this was unproven prior to implementation [3]. Ideally, thorough testing should be carried out before interventions are implemented [32]; this could have compromised the design of the current study as well as limiting campaign effectiveness nationally. The campaign was reported to be based on SM concepts [3], but is unclear from the marketing strategy whether adherence to SM criteria was measured. It is also unclear whether the campaign additionally drew on the behaviour change literature. There is evidence that basing behaviour change interventions on psychological theory improves outcome [33], so the lack of a theoretical basis may have limited the intervention’s impact. The aim of Change4Life was to prevent obesity, but no mention was made of ‘obesity’ in any of the campaign materials [4]. It has been argued that this was contrary to the evidence base on which it was developed, which was clearly to prevent obesity, and perhaps this reduced its impact since increasing knowledge about obesity without mentioning it is problematic [34].

The fact that the intervention appeared to have negative effects on attitudes and behaviour in higher SES families is difficult to explain. The qualitative findings suggested that some of these families found the information patronising, and they may have used the research questionnaire as an opportunity to express their displeasure with the campaign. Given that C4L primarily targeted lower SES families [3], it is not surprising that the materials did not appear relevant to those who were more educated. This raises the importance of ensuring that messages reach the intended target audience. There were some encouraging effects in lower SES families who actively engaged with the intervention. However, in the qualitative interviews, even lower SES families considered their current eating and activity behaviours to be satisfactory, so the materials had not convinced them of the need for change, although several barriers were raised by lower SES parents (e.g. time to engage with monitoring, cost, unrealistic changes), which implied that they might like to change if it were easier.

### Strengths and limitations

C4L was launched in January 2009, and study recruitment began in the summer of 2009, so contamination of the control group is a potential issue. This was reflected in the high awareness ratings at baseline. However, only two people completed and returned their HTK questionnaire outside of the study time-frame which suggests that although the majority of families had been exposed to the campaign, virtually none had actively engaged with it. There were differences between parents who returned the follow-up questionnaire and those who did not; the former were younger and more likely to be white and university educated. They also rated diet and activity as more important but interestingly perceived their child’s diet and level of activity to be adequate, although they reported finding it less easy to ensure that their child ate healthily or was physically active. This suggests that there was response bias for the follow-up questionnaire resulting in those included in the main analyses being of higher SES and generally more interested in diet and physical activity.

Only a third of those invited to participate in the study returned a baseline questionnaire. Response rates in other evaluations of social marketing campaigns varied from approximately 20% to over 60% [27,35], but these typically used telephone surveys. While this response rate was modest, it was comparable to some evaluations and the sample was large and demographically diverse. All data were self-reported, although validated questionnaires were used where possible and where these were not available, test-retest validation indicated adequate reliability. Brief questionnaires were used to assess children’s diet and physical activity to maximise participation, however, there are clearly limitations of doing this. There are also limitations in the monitoring and modelling questions since these did not cover all of the dietary and activity targets in C4L; these were not adapted however because they were taken from validated and widely used questionnaires. The use of a randomised controlled study design allowed for comparison with a ‘usual care’ control group.

The study only evaluated a single component of the C4L campaign, the ‘family information pack’ (either with or without personalised information), therefore it was not an evaluation of the full campaign. Adherence to SM principles was not included as an outcome in the current study since this was outside the study’s remit as defined by the funder. In addition, the HTK intervention tested here did not exactly replicate how the ‘family information pack’ was intended; we sent it to all participants whereas it was designed for voluntary uptake. Also, obesity prevention behaviour is likely to comprise slow changes and requires a cultural shift, one of the campaign’s aims. Full evaluation of these broad effects would require a broader range of outcomes, including impacts on society (e.g. environmental factors such as healthy food accessibility, and access to affordable leisure facilities) and a long follow-up period. Despite these limitations, this study is the only systematic evaluation of any elements of C4L, therefore is of importance for policy makers and researchers.

## Conclusions

This study indicates that although awareness of C4L was high, the campaign materials had little impact on attitudes or behaviours. Part of this may be because parents on the whole did not want to change or engage with the intervention, but rather viewed it as ‘for the kids’. There were some interesting differences in how lower and higher SES groups reacted to the intervention, with the latter showing some evidence of adverse effect. This highlights the need for social marketing campaigns to reach their ‘target group’. It is possible that there were wider benefits of the campaign, which were not captured in the outcomes used in this study, through obesity issues being raised up the national agenda. These disappointing findings suggest that future social marketing campaigns in the childhood obesity area should focus on a smaller range of behavioural targets, be specific about who is being targeted (i.e. parent vs. child), use behaviour change theory to inform intervention design, and carry out more formal pilot testing. Because so few studies have evaluated obesity prevention campaigns, this is an important area to develop measures and methods to enhance our understanding of their effects.

## Endnotes

^a^We use the term parents to include carers for economy of space.

## Competing interests

The authors declare that they have no competing interests.

## Authors’ contributions

HC was involved with study planning/ design, supervised data collection, conducted the analyses, and drafted the manuscript. RL was involved in study planning/design and data collection. JW conceived of the study, participated in study design, provided overall supervision for the trial and contributed to writing the manuscript. All authors read and approved the final manuscript.

## Pre-publication history

The pre-publication history for this paper can be accessed here:

http://www.biomedcentral.com/1471-2458/12/404/prepub

## Supplementary Material

Additional file 1 Table S1.Outcome measures used in the study questionnaire. Click here for file
