# Supplementary material for: Cluster-randomised trial to evaluate the ‘Change for Life’ mass media/ social marketing campaign in the UK
Source: BMC Public Health. 2012 Jun 6;12:404. doi: 10.1186/1471-2458-12-404 (PMC3541256; doi:10.1186/1471-2458-12-404)
Supplement: Additional file 1 — Table S1.Outcome measures used in the study questionnaire. [file 1471-2458-12-404-S1.pdf]

Supplemental Table: outcome measures used in the study questionnaire

Outcome measures used in evaluation study	Response options	Scoring and interpretation
**Parent attitudes and intention to change child behaviours**		
Rating of importance/ ease/ intention to change[1]:<br>Limit high-fat foods eaten at mealtimes<br>Limit number of sugary drinks<br>Avoid sugary breakfast cereals<br>Snack mainly on healthy foods<br>Not to eat too much food<br>Eat 5 or more portions of fruit and vegetables<br>Eat 3 meals at regular times<br>Eat no more than 2 snacks<br>Spend at least 60 minutes being active<br>Limit time spent watching TV/ on computer	Each item on a five-point Likert scale (‘not important’ to ‘very important’; ‘difficult’ to ‘easy’; ‘definitely do not intend to do’ to ‘definitely intend to do)	Each item had a possible score of 1-5, a higher score indicated greater importance, ease and rating of intention to change
**Child behaviour**		
Intake of[1]:<br>Snacks<br>Sugary drinks<br>Fruits<br>Vegetables	Frequency that child typically has each food and drink (‘less than once a week’, 1 a week’, ‘2-3 times a week’, ‘4-6 times a week’, ‘1 a day’, ‘2 a day’, and ‘3 or more a day’)	Each item had a possible score of 1 to 7, mean score calculated to create an overall healthy eating score (snack and sugary drink items reversed). A higher

		score indicated healthier eating.
Number of days child is physically active for at least 60 minutes in a typical week[1]<br>(This did not need to be in one go and a definition of physical activity with examples was given)	Number of days child is active ('less than 1 day a week', '1 day a week', '2 days a week', '3 days a week', '4 days a week', '5 days a week', and '6 or more days a week').	For analysis, an average of the daily hours of activity was calculated.
Hours of TV/ video/ computer on a typical weekday/ weekend day (including evenings)[1]	Media viewing in a typical weekday and weekend day ('none', less than 1 hour a day', '1-2 hours a day', '2-3 hours a day', '3-4 hours a day', and 'more than 4 hours a day').	For analysis, an average of the daily hours of viewing was calculated.
**Parent behaviour**		
Monitoring of food intake[2]:<br>Snack foods (e.g. crisps, cheesy crackers)<br>High fat foods<br>Sweet things (e.g. sweets, ice-cream, cake, biscuits, chocolate)	Frequency that parent keep track of each food type ('never', 'rarely', 'sometimes', 'often', and 'always').	A higher mean score indicated more monitoring.
Modelling healthy eating by[3]:<br>Eating healthy foods<br>Eating healthy foods in front of child even if not favourite	Parents' agreement over doing each behaviour ('disagree', 'slightly disagree', 'slightly agree', and 'agree').	A higher mean score indicated more modelling.

Showing enthusiasm about healthy foods<br>Showing how much enjoy healthy foods		
Monitoring of physical activity[4]	Frequency that parent tracks how much physical activity child does (‘never’, ‘rarely’, ‘sometimes’, ‘often’, and ‘very often’).	A higher score indicated more monitoring
Modelling of being active[5]:<br>Being active<br>Being active in front of child even if prefer not to<br>Showing enthusiasm about being active<br>Showing how much I enjoy being active	Parents’ agreement over doing each behaviour (‘disagree’, ‘slightly disagree’, ‘slightly agree’, and ‘agree’).	A higher mean score indicated more modelling.

[1]Questions developed for study; [2]Taken from the Child Feeding questionnaire (Birch et al, 01); [3]Taken from the ‘Comprehensive Feeding Practices Questionnaire’ (Musher-Eizenman & Holub, 07); [4]Adapted from the PACE physical activity measure for adolescents (PACE, 01); [5]Adapted for physical activity from the ‘Comprehensive Feeding Practices Questionnaire’ (Musher-Eizenman & Holub, 07)
